# Dendritic Cell Subsets and Effector Function in Idiopathic and Connective Tissue Disease-Associated Pulmonary Arterial Hypertension

**DOI:** 10.3389/fimmu.2019.00011

**Published:** 2019-01-22

**Authors:** Denise van Uden, Karin Boomars, Mirjam Kool

**Affiliations:** Department of Pulmonary Medicine, Erasmus MC, Rotterdam, Netherlands

**Keywords:** dendritic cell, dendritic cell subsets, pulmonary arterial hypertension, idiopathic pulmonary arterial hypertension, autoimmune disease, dendritic cell effector function, connective tissue disease

## Abstract

Pulmonary arterial hypertension (PAH) is a cardiopulmonary disease characterized by an incurable condition of the pulmonary vasculature, leading to increased pulmonary vascular resistance, elevated pulmonary arterial pressure resulting in progressive right ventricular failure and ultimately death. PAH has different underlying causes. In approximately 30–40% of the patients no underlying risk factor or cause can be found, so-called idiopathic PAH (IPAH). Patients with an autoimmune connective tissue disease (CTD) can develop PAH [CTD-associated PAH (CTD-PAH)], suggesting a prominent role of immune cell activation in PAH pathophysiology. This is further supported by the presence of tertiary lymphoid organs (TLOs) near pulmonary blood vessels in IPAH and CTD-PAH. TLOs consist of myeloid cells, like monocytes and dendritic cells (DCs), T-cells, and B-cells. Next to their T-cell activating function, DCs are crucial for the preservation of TLOs. Multiple DC subsets can be found in steady state, such as conventional DCs (cDCs), including type 1 cDCs (cDC1s), and type 2 cDCs (cDC2s), AXL^+^Siglec6^+^ DCs (AS-DCs), and plasmacytoid DCs (pDCs). Under inflammatory conditions monocytes can differentiate into monocyte-derived-DCs (mo-DCs). DC subset distribution and activation status play an important role in the pathobiology of autoimmune diseases and most likely in the development of IPAH and CTD-PAH. DCs can contribute to pathology by activating T-cells (production of pro-inflammatory cytokines) and B-cells (pathogenic antibody secretion). In this review we therefore describe the latest knowledge about DC subset distribution, activation status, and effector functions, and polymorphisms involved in DC function in IPAH and CTD-PAH to gain a better understanding of PAH pathology.

## Introduction Pulmonary Arterial hypertension

Pulmonary arterial hypertension (PAH) is characterized by a mean pulmonary arterial pressure (PAP) of ≥25 mmHg at rest and a mean capillary wedge pressure of ≤15 mmHg (1). The high PAP causes hypertrophy of the right ventricle (RV) leading eventually to RV dilatation, heart failure, and ultimately death. Particularly small pulmonary arteries (PAs) and arterioles are affected. They show a thickened vascular wall and formation of plexiform lesions due to endothelial dysfunction and proliferation of all three cell layers, the endothelium, smooth muscle cells (SMC), and the adventitia (2).

PAH patients can be subdivided into groups based on associated conditions and risk factors. However, in a substantial proportion of PAH patients no cause or associated condition can be identified: idiopathic PAH (IPAH). In another subgroup of patients, PAH is associated with autoimmune diseases (AD) such as connective tissue disease (CTD). CTD includes systemic sclerosis (SSc), systemic lupus erythematosus (SLE), rheumatoid arthritis (RA), and mixed connective tissue disease (MCTD). SSc is the most common AD associated with PAH, followed by SLE (3–6). PAH patients have a low 1-year survival rate: only 82% of SSc-PAH patients and 93% of IPAH patients are still alive after 1 year (6).

## Role for Immune Activation in the Development of PAH

The presence of PAH in a proportion of autoimmune patients suggests that activated immune cells (or their mediators) directly provoke pulmonary vascular remodeling. Local immune activation is also observed as tertiary lymphoid organs (TLOs or ectopic lymphoid structures) are present in the lungs of IPAH and CTD-associated PAH (CTD-PAH) patients (7, 8). TLOs are organized structures similar to lymph nodes (LNs), including distinct T-cell areas containing dendritic cells (DCs), organized B-cell follicles with germinal centers (GCs), high endothelial venules (HEV), and lymphatics. TLOs most likely develop due to long-lasting local immune activation and are considered a hallmark of chronic disease (9). In lungs of IPAH patients, TLOs are found in the vicinity of PAs, suggesting that they promote vascular remodeling (7). Not surprisingly, as TLOs are characteristic for ongoing/chronic immune activation, they are often found in target organs of several ADs. For instance, in SLE patients TLOs are present in the kidneys, and in SSc-PAH patients TLOs have even been found in the lungs (8, 10, 11). Even though the SSc-PAH patient group used in this study is small, it is conceivable that TLOs are present in the lungs of various CTD-PAH patients. In addition, it is very likely that immune activation in PAH patients will also occur in draining LNs.

During chronic antigenic stimulation, the lymphotoxin (LT)α1β2-LTβ receptor axes is crucial for development of TLOs (12), whereby lymphoid tissue inducer (LTi) cells interact with lymphoid tissue organizer (LTo) cells. Repeated DC injection in the lungs of mice, mimicking chronic activation, provokes TLO development (13). Activated DCs can produce chemokines which attract T-cells and B-cells (e.g., CCL19/21 and CXCL13, respectively), as well as T- and B-cell survival factors (e.g., interleukin (IL)-15 and BAFF/IL-6, respectively) (13–17). They furthermore secrete cytokines creating a pro-inflammatory milieu and promote innate and adaptive responses. This milieu can also induce post-translational modifications of proteins, altering self-antigens into new antigens which could provoke autoimmune responses as seen in SLE (18). Within TLOs and LNs, tissue-migrated DCs present antigens to naïve T-cells, inducing their activation and differentiation. The main T helper (Th)-cell subsets are Th1, Th2, Th17, follicular Th-cells (Tfh), and regulatory T-cells (Tregs). Within the GC reaction in TLOs and LNs, Tfh-cells provide help to B-cells by producing cytokines that induce class switching, survival, proliferation, and antibody production.

The role of DC subsets and their effector function in pathogenesis of IPAH, AD, and CTD-PAH will be discussed in this review and is shown in Table 1.

**Table 1:** Involvement of DCs and monocytes in IPAH, AD, and CTD-PAH.

	**Disease**	**Major finding**	**Tissue**	**References**
cDC	IPAH SLE	cDCs are decreased in proportion and number	Blood	(19–23)
	SSc	cDC2s produce more IL-6, IL-10 and TNFα after TLR2 and TLR4 stimulation	Blood	(24, 25)
	SSc-PAH	•A *TLR2* polymorphism in AD patients is associated with PAH development •cDCs carrying this *TLR2* polymorphism produce more cytokines (e.g., IL-6)	Blood	(26)
	IPAH	cDCs numbers are increased	Lung	(27)
	IPAH AD^a^	cDCs are present in TLOs in target organs	Lung, Thyroid tissue	(7, 28)
pDC	IPAH	The number of pDCs is unaltered	Blood	(27)
	SLE SSc	pDCs are decreased in proportion and number	Blood	(22, 23, 29)
	SSc	pDCs predominantly secrete CXCL4	Blood, Skin	(30)
	IPAH	•pDC numbers are increased •pDCs are located around pulmonary vessels	Lung	(27)
	SLE SSc	pDCs are increased in diseased tissue	Skin	(29, 31)
Monocytes and mo-DCs	IPAH	hyporesponsive monocytes to TLR4 stimulation	Blood	(32)
	SSc-PAH	Monocytes show an activated profile (mRNA expression)	Blood	(33)
	SSc SSc-PAH	The number of non-classical monocytes is increased	Blood	(34)
	SSc	CXCL10, CXCL8, and CCL4-producing non-classical monocyte subset is increased	Blood	(24)
	IPAH	Monocytes have either a similar or decreased activation status, depending on the study	Blood	(19, 35)
	IPAH	*In vitro* generated mo-DCs have either an increased or decreased Th-cell stimulatory capability, depending on the study	Blood	(19, 35)
	SSc	mo-DCs carrying the *TLR2* polymorphism produce more cytokines (e.g., IL-6)	Blood	(26)
	IPAH	CD14+ cells are increased around pulmonary arteries	Lung	(36)

a *Graves disease and Hashimoto's thyroiditis, cDC, conventional dendritic cell; pDC, plasmacytoid dendritic cell; mo-DC, monocyte-derived-dendritic-cell; PAH, pulmonary arterial hypertension; IPAH, idiopathic pulmonary arterial hypertension; AD, autoimmune disease; CTD-PAH, connective tissue disease-associated PAH; SLE, systemic lupus erythematosus; SSc, systemic sclerosis; TLO, tertiary lymphoid organ; PAs, pulmonary arteries; TLR, toll-like receptor*.

## Dendritic Cells in IPAH, CTD-PAH, and AD

DCs are equipped with pathogen recognition receptors (PRRs) like toll-like receptors (TLR) to sense their surroundings. Antigen recognition leads to DC activation and migration toward LNs. Activated DCs upregulate co-stimulatory molecules like CD86, produce pro-inflammatory cytokines, and present antigen to T-cells using major histocompatibility complex class-II (MHC-II). In TLOs, DCs are mature, indicated by high CD86 expression and IL-12 production (37). The maintenance of TLOs in two lung infection models, has been shown to be dependent on DCs as they disintegrate when DCs are ablated (13, 38). Furthermore, impaired DC migration due to defects in the CCR7-signaling, has been shown to lead to the formation of bronchus-associated lymphoid tissue (39).

Under steady state conditions, several DC subsets with unique functions can be identified (40, 41). Conventional DCs (cDCs), identified by CD11c, and HLA-DR expression in humans, are a major DC subset and can be divided in two subtypes, type 1 cDCs (cDC1s) and type 2 cDCs (cDC2s). cDC1s express IRF8 and CD141 and excel in cross presentation (42). IRF4 and CD1c classify cDC2s, which are potent inducers of Th-cell responses. Plasmacytoid DCs (pDCs) produce interferons (IFN) and do not express CD11c, but express HLA-DR and CD123. Recently, within this HLA-DR^+^CD123^+^ population potent Th-cell inducers have been found, which additionally express AXL and Siglec6 (AXL^+^Siglec6^+^ (AS)-DCs) (43, 44). Under inflammatory conditions monocytes can differentiate into DCs, giving rise to monocyte-derived-DCs (mo-DCs).

### Conventional Dendritic Cells

**Figure 1:**
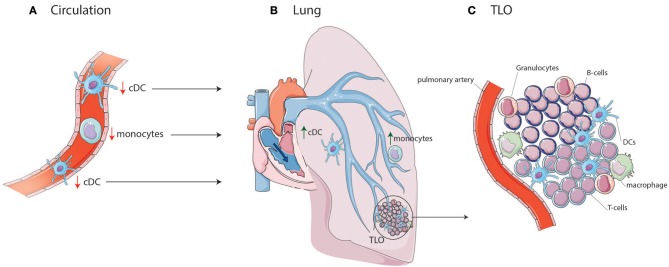
cDC and monocyte migration toward lung TLOs. **(A)** cDCs and monocytes are decreased in circulation of IPAH patients due to migration to the lungs in which cDCs and monocytes are increased. **(B)** In the lung they can add to the development of TLOs surrounding PAs. **(C)** TLOs consist, besides DCs, of different immune cells such as T-cells, B-cells, macrophages, and granulocytes.

In IPAH patients, the proportion of circulating cDCs is decreased compared to controls (19). Numbers of circulating cDCs are also altered in several ADs associated with PAH. Both cDC1s and cDC2s are decreased in proportion and number in SLE patients compared to HCs, especially in patients with active disease (20–23). The decrease in circulating cDCs in PAH could indicate an increased cDC migration toward lung TLOs (Figure 1). In support of this idea, DCs can be found in lung TLOs of IPAH patients and cDC numbers were increased in total lung cell suspensions of these patients (7, 27). In IPAH TLOs, DCs are found inside T-cell zones, suggesting that they promote T-cell activation. In patients with ADs, cDCs in TLOs show increased expression of costimulatory molecules and a cDC2 phenotype, since they express CD1c and CD11c (28). Alternatively, the reduction in circulating cDCs might also be caused by alterations in cDC viability or DC progenitors resulting in a decreased output of cDCs from the bone marrow.

In addition to DC or DC precursors entering the affected tissue from the blood circulation, DCs may accumulate in tissue and contribute to TLO formation as they fail to go to LNs (39). Upon activation, DCs upregulate CCR7. The CCR7 allows the DC to respond to CCL19 and CCL21 expressed by the lymphatic endothelial cells and to enter the lymphatic vessels to migrate to the draining LN. Both CCL19 and CCL21 are expressed by lymphatic vessels in IPAH patients, which could facilitate DC attraction (7). Strikingly, CCR7-deficient mice develop lung TLOs and signs of PH, perhaps due to DC retention in the lungs (39, 45). DCs, amongst other cells, can produce CCL20 and CXCL13, which attract T-cells, B-cells, and immature DCs. CCL20 and CXCL13 mRNA expression are increased in IPAH lungs compared to controls (7), contributing to TLO formation. However, the cell responsible for this increased expression in IPAH is yet unknown.

Research into cDC subset activation is still limited in PAH and ADs. In SSc patients, circulating cDC2s produce more IL-6, IL-10, and TNF-α after TLR2 and TLR4 stimulation (24, 25). These cytokines appear to play a central role in the immunopathology of PAH, as IL-6 and IL-10 are increased in the serum of IPAH patients and correlate with mortality (46). Especially IL-6 appears to be a crucial cytokine in PAH pathobiology, as mice overexpressing IL-6 develop signs of PH, while IL-6-deficient mice do not develop PH after hypoxia (47, 48). At this time, a phase II trial using Tocilizumab, an IL-6 receptor antagonist, is conducted in PAH patients (49).

**Figure 2:**
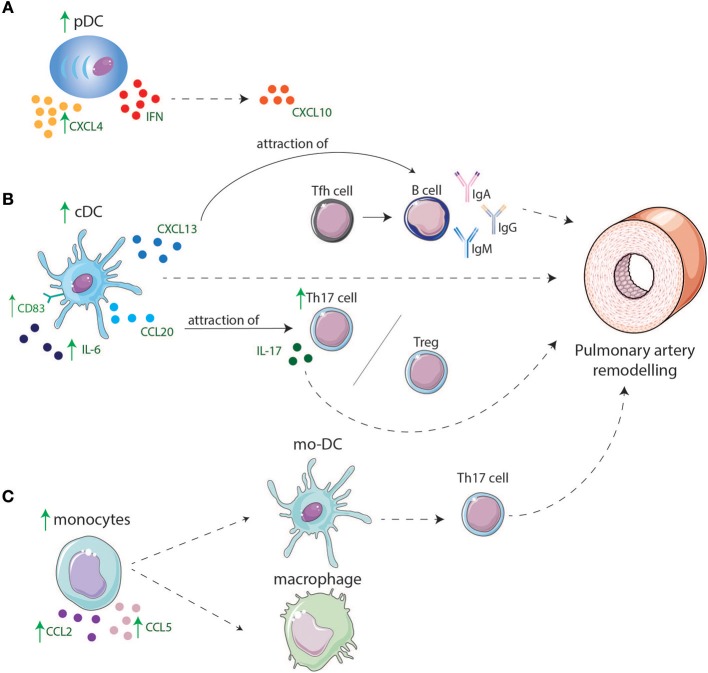
Involvement of DCs and monocyte in lungs of IPAH and CTD-PAH patients. **(A)** pDCs are increased in lungs and might play a role in IPAH and CTD-PAH pathology by producing higher levels of CXCL4 and CXCL10 that is induced by IFNs. **(B)** cDC display higher levels of CD83 and have an enhanced cytokine production e.g., IL-6. cDCs are increased in lungs of PAH patients and can directly lead to PA remodeling or indirectly by production of CXCL13 and CCL20. CXCL13 leads to migration of B-cells toward the lungs, B-cells will produce pathogenic antibodies after interaction with Tfh cells, leading to remodeling of PAs. CCL20 attracts T-cells such as Tregs and Th17 cells leading to an increase in Th17 cells in the lung resulting in a Th17/Treg disbalance and by IL-17 production contributes to PA remodeling. **(C)** Monocytes are increased in the lung and produce CCL2 and CCL5 which might lead to attraction of other monocytes. Monocytes might differentiate in macrophages or mo-DCs. Mo-DCs induce Th17 cells adding to PA remodeling.

In conclusion, in both IPAH and ADs circulating cDC proportions are decreased possibly due to migration to target organs, where they can both initiate adaptive immune responses and maintain TLOs (Figure 2B). Currently, only little is known about cDC subset distribution and function in IPAH, CTD-PAH, and ADs.

### Plasmacytoid Dendritic Cells

Plasmacytoid DCs are predominantly found in lymphoid tissues and blood in steady state conditions. During inflammation, pDCs home toward peripheral tissues, produce type I IFNs, and promote activation of immune cells. In IPAH lungs pDC numbers are enhanced and pDCs are specifically located around the pulmonary vessels, while circulating pDC numbers are unaltered (27). In contrast, in SLE and SSc patients, circulating pDC number and frequency are decreased compared to controls, which could be due to emigration into diseased tissues (22, 23, 29, 31). Indeed, pDCs are present in diseased organs of SSc patients (29). Several ADs are associated with the interferon gene signature (IGS), to which different cells contribute. pDCs are major contributors to the IGS through their production of type I IFNs. One of the most strongly upregulated genes in pDCs within the IGS is CXCL10 (50). Augmented serum CXCL10 levels are associated with PAH in SSc patients (51). Likewise, in IPAH patients, serum CXCL10 is elevated and even associated with poor RV function (52), suggesting the possibility of a prominent role for pDCs in disease immunopathology. Next to IFNs, pDCs are also large producers of CXCL4 in SSc (30). CXCL4 can induce an influx of CD45^+^ cells in target tissues, perhaps leading to tissue remodeling and disease progression.

The associations of pDC with CTD-PAH and the increase in pDCs in lungs of IPAH patients suggest that type-I IFN and chemokine secretion by pDCs not only play an important role in several ADs, but also in CTD-PAH and IPAH pathology (Figure 2A).

### Monocytes and Monocyte-Derived DCs

Monocytes are precursors of mo-DCs that arise under inflammatory conditions (40). Monocytes are heterogeneous and can be divided into 3 subsets based on CD14 and CD16 expression (53, 54). Classical monocytes, also called inflammatory monocytes, express CD14 and can infiltrate tissues, produce pro-inflammatory cytokines, and differentiate into inflammatory macrophages. Classical monocytes express several PRRs and are superior in phagocytosis. Monocytes expressing both CD14 and CD16 are termed intermediate monocytes, can also produce pro-inflammatory cytokines (55) and are unique in their ability to produce reactive oxygen species. Their gene expression signature indicates their ability to present antigens and induce T-cell activation (56). Intermediate monocytes specifically promote pro-inflammatory Th17-cell responses, which also contribute to PAH development, as discussed below (55). Finally, non-classical monocytes, expressing CD16, are known to survey the endothelium for danger signals (54). They differentiate into tissue-resident macrophages in steady state or into anti-inflammatory macrophages during inflammation, to repair damaged tissues.

The number of non-classical monocytes is increased in SSc associated with PAH development, whereas there is no difference in the number of classical monocytes (34). The number of CTD-PAH patients in this study was very small, so this should be confirmed in a larger cohort. Increased numbers of CD14^+^ cells, including classical/intermediate monocytes and macrophages, are observed around PAs of IPAH patients (36). Monocytes might be attracted to the PAs through their expression of CCR2 and CCR5 and an increased expression of their ligands CCL2 and CCL5 in lungs and serum of IPAH patients (57, 58). In SSc and CTD-PAH enhanced CCL2 is also observed in either skin or serum (59–61).

Strikingly, circulating monocytes of IPAH patients are hyporesponsive, as demonstrated by decreased cytokine production upon TLR4 stimulation (32). The local and/or systemic pro-inflammatory milieu in IPAH patients could provoke a feedback mechanism, resulting in hyporesponsive monocytes. However, the underlying mechanism is still unknown and further research is needed. In contrast to IPAH monocytes, monocytes from SSc-PAH patients are activated, as shown by their mRNA expression profile. This profile is even discriminative between SSc-PAH and SSc patients (33). Non-classical monocytes, expressing CXCL10, CXCL8, and CCL4 are involved in SSc pathology, and are found in increased numbers in SSc patients compared to controls (24).

Mo-DCs for *in vitro* assays, used to model and monitor human DC function, are commonly generated from monocytes. Contradictory results have been found using this model in IPAH. Decreased activation of monocytes together with lower T-cell stimulation (19), as well as a similar activation status with an increased Th-cell stimulatory capability have been observed (35). These opposite findings might be caused by the type of stimulation used to mature mo-DCs and different mo-DC:T-cell ratios in the T-cell stimulation assays.

Taken together, increased pulmonary expression of chemokines may attract monocytes to lungs of IPAH and CTD-PAH patients, where they become activated and alter their gene expression due to the pro-inflammatory environment. These altered monocytes may give rise to mo-DCs, which arise at places of inflammation and can induce T-cell activation (Figure 2C).

## Effector Function of DCs in IPAH, CTD-PAH and ADS

### T-Cell Responses

DCs excel at antigen presentation to T-cells and together with their costimulatory molecule expression and cytokine production, they are pivotal for the succeeding T-cell response. Specifically, Th17-cells are implicated in the pathogenesis of many ADs and are observed inside mature TLOs of IPAH patients (7). Th17 differentiation from naïve Th-cells occurs in the presence of IL-1β, IL-6, and TGFβ (62), cytokines produced by activated DCs. Both IL-1β and IL-6 are elevated in serum of IPAH patients (46). Th17-cells are the main source of IL-17, IL-21, and IL-22. IL-21^+^ cells are present in remodeled PAs of IPAH patients (63). In addition, IL-17 may affect structural remodeling observed in PAH, as IL-17 enhances fibroblast proliferation and collagen production *in vitro* (64). In SSc, IL-17 induces adhesion molecule expression and IL-1/chemokine production on endothelial cells (ECs) (65–67). Additionally, in IPAH PBMCs the IL-17 gene is hypo-methylated, indicating increased IL-17 transcription and supporting a possible role for Th17-cells in the pathology of IPAH (35). Indeed, IL-17 gene expression is enhanced in lungs of both IPAH and SSc-PAH compared to idiopathic pulmonary fibrosis (IPF) and pulmonary fibrosis associated SSc (SSc-PF) (68), this IL-17 may be expressed by cells in TLOs as well as in tissues outside of TLOs.

Furthermore, IL-23, also produced by DCs, stabilizes the phenotype of Th17-cells, but also promotes their pro-inflammatory potential (62). Th17-cells are also highly plastic cells and under the influence of IL-23 start co-expressing cytokines from the Th1-cell lineage. This leads to possibly pathogenic IFNγ-producing Th17-cells, also called Th17.1-cells. Enhanced expression of the IL-23 receptor on Th17(.1)-cells might contribute to their pro-inflammatory pathogenic phenotype (62, 69, 70). IL-23 is increased in exhale breath condensate of SSc patients, so perhaps Th17 plasticity plays a role in SSc pathology (71). Furthermore, IFNγ, IL-12, and TNFα can induce plasticity toward Th17.1-cells (62). Both serum IL-12 and TNFα are enhanced in IPAH patients and mRNA transcripts of these cytokines were increased in lungs rats in a PH model (46, 72). IL-17/IFNγ-double producing Th-cells are observed within the arteries of atherosclerosis patients, where they provoke pro-inflammatory cytokine production (e.g., IL-6, CXCL10) by vascular SMCs (73). This feedback loop could also exist within PAH, since IL-6 is highly produced by pulmonary ECs of IPAH patients. In addition, IL-6 promotes SMC proliferation in a hypoxia-induced PH model (74, 75). Blocking of IL-6 signaling improved PH physiology in a hypoxia-induced PH mouse model and prevented accumulation of Th17-cells (63). IL-6 also converts Th17-cells into IL-17+ Tregs, which are less suppressive than conventional Tregs (76). In SSc, IL-17+ Tregs are observed in the circulation and possibly also in the skin, indicated by IL-17 and FoxP3 positivity (64, 65, 77). The balance between pro-inflammatory Th17-cells and anti-inflammatory Tregs is crucial to control autoimmune features. IL-6 is a key cytokine in Th17/Treg balance, since TGF-β alone polarizes naïve Th-cells to Tregs, while TGF-β together with IL-6 induces Th17-cells (78). Active TGF-β signaling is very prominent in PAH and can be produced by different cells, like monocytes and DCs (79). However, whether DC-derived IL-6 plays a prominent role is unknown yet, as many cells can produce IL-6. In favor of a disturbed balance are the decreased number of Tregs observed in SLE, which correlates with disease severity (66). In CTD-PAH patients Th17-cells and Th17-related cytokines are elevated compared to AD patients without PAH (80). The disturbed Th17/Treg ratio even appears to correlate with PAH severity in APAH patients (80). This demonstrates that Th17-cells and Tregs are implicated not only in ADs but also in PAH (80).

Therefore, Th17 plasticity and Th17/Treg balance may contribute to ADs and PAH, potentially in part by modulating vascular remodeling.

### Humoral Immune Response

Apart from their interaction with Th17-cells, DCs can induce (immature) Tfh-cells, which develop under the influence of IL-21, IL-6, IL-12, and IL-27 (78). In mature TLOs containing GCs, Tfh-cells interact with B-cells, leading to either antibody-producing plasma cells or memory B-cells. There is clear evidence for B-cell dysregulation in IPAH and CTD-PAH (81, 82). In IPAH patients circulating B-cells have an increased expression of genes involved in inflammatory mechanisms, host defense, and endothelial dysfunction, suggesting increased activation of B-cells (82). Also numbers of circulating plasmablasts are elevated in IPAH patients (83). Anomalies in B-cell homeostasis were also observed in SSc-PAH patients, with increased circulating IgD+ B-cell proportions (81). Tfh-cell numbers crucially control the development of auto-reactive B-cells, since an increase in Tfh-cell number can lead to increased autoantibody production (84, 85). In several ADs, Tfh-cells are increased in blood and target organs (86–89). Serum IgG, IgM, and IgA antibodies are elevated in IPAH patients, and EC-specific IgA promotes cytokine production and upregulation of adhesion molecules (83, 90–92). IgG and IgM antibodies directed against EC-surface antigens are also found in ADs and CTD-PAH, being most prevalent in SSc-PAH patients, followed by IPAH patients and SSc patients without PAH (92). IgG antibodies in SSc and SLE were directed against microvascular ECs antigens, while IgG in SSc, IPAH, and CTD-PAH recognized microvascular dermal and lung EC antigens, and vascular SMCs (90, 91, 93–95). Auto-reactive IgG provoked EC dysfunction, induced pro-inflammatory signals, and increased adhesiveness of T-cells to ECs, which also modulated migration and proliferation of SMC. These autoantibodies from SSc or CTD-PAH patients can directly cause signs of PH when injected into healthy mice (96). It is unknown where the autoantibodies found in IPAH and CTD-PAH patients are produced. TLO might be a likely location since Tfh-cells and B-cells, and perhaps antigens, are present in these TLOs. However, these autoantibodies can also be produced in the draining LNs.

In brief, pathogenic autoantibodies in CTD-PAH and IPAH might be produced by dysregulated B-cells that interact with Tfh-cells in TLOs. These autoantibodies recognize protein epitopes expressed by ECs, leading to endothelial dysfunction and vascular remodeling. So far, the role of Tfh-cells in IPAH is unknown and further research is needed.

## Genetics

Increased activation of the immune system in PAH is also supported by different polymorphisms observed in genome wide association studies. A polymorphism in *TLR2* of SSc patients is associated with PAH development (26). Functional analysis of mo-DCs and cDCs carrying the *TLR2* polymorphism showed enhanced cytokine production, including IL-6, compared to control DCs. As discussed above, IL-6 plays a prominent role in PAH pathology. Strikingly, a decreased IL-6 serum level was observed in healthy individuals and patients with a single nucleotide polymorphism in the promotor region of the *IL-6* gene, *IL-6-572C/G*, which correlated with decreased risk to develop IPAH (97). SNPs might not only be useful to determine disease susceptibility but also to determine disease onset or activity, as is seen for a specific SNP in *TGFB* gene in heritable PAH patients carrying a BMPR2 mutation (98). Another genetic association found in both PAH and SSc involving immune activation is a SNP in the *TNFAIP3* gene (99). *TNFAIP3* encodes for the ubiquitinating enzyme A20, which is crucial for down-regulation of the nuclear factor-kappa B (NF-κB) signaling pathway and thereby cell activation (100). Macrophages, pulmonary arterial ECs, and pulmonary arterial SMCs in end-stage IPAH patients showed an increased expression in NF-κB (101), suggesting an important role for the NF-κB pathway in IPAH.

This demonstrates that several SNPs and genes that are involved in DC function are present in PAH patients.

## Future Directions

In conclusion, different DC subsets are involved not only in the pathobiology of ADs but appear to play a role in the pathobiology of IPAH and CTD-PAH as well. However, the exact role of these DCs in PAH development has not been fully elucidated. The increasing knowledge on DC biology obtained by advanced immunological techniques has led to a more unified method to identify DC subsets and the discovery of new DC subsets. Determining the role of all currently known DC populations, including AS-DCs, as well as their specific functions may help to unravel the pathobiology of PAH. This might lead to new opportunities for therapies targeting specific DC subsets, their activation, and/or their effector function.

## Author Contributions

DvU and MK wrote the manuscript. KB contributed to the review of the manuscript. All authors approved the manuscript for publication.

### Conflict of Interest Statement

The authors declare that the research was conducted in the absence of any commercial or financial relationships that could be construed as a potential conflict of interest.

## Abbreviations

PAH: Pulmonary arterial hypertension
PAP: pulmonary arterial pressure
RV: right ventricle
PAs: pulmonary arteries
SMC: smooth muscle cell
IPAH: idiopathic PAH
CTD: connective tissue diseases
CTD-PAH: CTD-associated PAH
AD: autoimmune disease
SSc: systemic sclerosis
SLE: systemic lupus erythematosus
RA: rheumatoid arthritis
MCTD: mixed connective tissue disease
SSc-PAH: Systemic sclerosis-PAH
TLOs: tertiary lymphoid organs
LNs: lymph nodes
DCs: dendritic cells
GCs: germinal centers
HEV: high endothelial venules
PH: pulmonary hypertensions
LT: lymphotoxin
LTi: lymphoid tissue inducers
LTo: lymphoid tissue organizer
Th: T helper
IL: interleukin
Tfh: follicular Th-cells
Tregs: regulatory T-cells
PRRs: pathogen recognition receptors
TLR: toll-like receptor
MHC-II: major histocompatibility complex class II
cDCs: conventional DCs
cDC1s: type 1 cDCs
cDC2s: type 2 cDCs
pDCs: Plasmacytoid DCs
IFN: interferons
AS–DCs: AXL^+^Siglec6^+^ DCs
mo-DCs: monocyte-derived dendritic cells
BM: bone marrow
IGS: interferon gene signature
PBMCs: peripheral blood mononuclear cells
LPS: lipopolysaccharide
ECs: endothelial cells
IPF: idiopathic pulmonary fibrosis
SSc-PF: pulmonary fibrosis associated SSc
NF-kB: nuclear factor-kappa B.
